# CRISPR/Cas genome editing in tomato improvement: Advances and applications

**DOI:** 10.3389/fpls.2023.1121209

**Published:** 2023-02-23

**Authors:** Jagesh Kumar Tiwari, Anand Kumar Singh, Tusar Kanti Behera

**Affiliations:** ^1^ Division of Vegetable Improvement, Indian Council of Agricultural Research-Indian Institute of Vegetable Research, Varanasi, Uttar Pradesh, India; ^2^ Division of Horticulture, Indian Council of Agricultural Research, Krishi Anusandhan Bhawan - II, Pusa, New Delhi, India

**Keywords:** abiotic stress, biotic stress, CRISPR/Cas9, plant architecture, flower, fruit quality, genome editing, tomato

## Abstract

The narrow genetic base of tomato poses serious challenges in breeding. Hence, with the advent of clustered regularly interspaced short palindromic repeat (CRISPR)-associated protein9 (CRISPR/Cas9) genome editing, fast and efficient breeding has become possible in tomato breeding. Many traits have been edited and functionally characterized using CRISPR/Cas9 in tomato such as plant architecture and flower characters (e.g. leaf, stem, flower, male sterility, fruit, parthenocarpy), fruit ripening, quality and nutrition (e.g., lycopene, carotenoid, GABA, TSS, anthocyanin, shelf-life), disease resistance (e.g. TYLCV, powdery mildew, late blight), abiotic stress tolerance (e.g. heat, drought, salinity), C-N metabolism, and herbicide resistance. CRISPR/Cas9 has been proven in introgression of *de novo* domestication of elite traits from wild relatives to the cultivated tomato and vice versa. Innovations in CRISPR/Cas allow the use of online tools for single guide RNA design and multiplexing, cloning (e.g. Golden Gate cloning, GoldenBraid, and BioBrick technology), robust CRISPR/Cas constructs, efficient transformation protocols such as *Agrobacterium*, and DNA-free protoplast method for Cas9-gRNAs ribonucleoproteins (RNPs) complex, Cas9 variants like PAM-free Cas12a, and Cas9-NG/XNG-Cas9, homologous recombination (HR)-based gene knock-in (HKI) by geminivirus replicon, and base/prime editing (Target-AID technology). This mini-review highlights the current research advances in CRISPR/Cas for fast and efficient breeding of tomato.

## Introduction

Tomato (*Solanum lycopersicum* L.) is one of the most economically important vegetable crops, which is consumed as fresh and processed products. Tomato is considered a functional and protective food because of its health-beneficial compounds such as vitamins A and C, minerals, and antioxidants mainly lycopene and beta-carotene (Causse et al., 2016). Breeding new tomato cultivars with current consumers’ demand and processing industrial requirements is very important, which needs wide genetic resources with target traits. Natural genetic variability has led to the development of many varieties with agronomic traits. Initially, induced mutagenesis caused by chemical or physical mutagens has been successfully applied in breeding, but it is quite labor-intensive, cumbersome, and time-consuming. Similarly, conventional breeding also rely upon phenotypic selection and requires a long breeding cycle. Hence, to overcome these issues the availability of genome sequence (900 Mb) allows functional genomics and rapid breeding *via* dissecting complex traits (Tomato Genome Consortium, 2012). Furthermore, tomato pan-genomes have been reported for 1000 accessions (Alonge et al., 2020) and 725 cultivated and wild species (Gao et al., 2019). Recently, progress in genomics-assisted breeding has been reviewed for accelerated tomato improvement (Hanak et al., 2022; Tiwari et al., 2022).

To address the issues faced with conventional breeding, chemical/physical mutagenesis and trangenics, plant breeding has become much easier and highly efficient while using the clustered regularly interspaced short palindromic repeat (CRISPR) and CRISPR-associated 9 protein (CRISPR/Cas9) genome editing tool. CRISPR/Cas9 is a powerful and precise genome editing technology that allows trait-specific targeted mutants and functional characterization of genes. CRISPR/Cas9 has been used to edit various traits in tomato (Rothan et al., 2019; Vu et al., 2020a; Chandrasekaran et al., 2021; Salava et al., 2021; Xia et al., 2021; Chaudhuri et al., 2022). Recently, CRISPR-edited GABA-rich tomatoes first entered the food market (Waltz, 2022). In this mini-review, we provide a brief overview of CRISPR/Cas9, its current application in tomato trait modifications, and research advances on sgRNA designing, cloning, transformation, and regulatory aspects.

## CRISPR/Cas9 genome editing technology

CRISPR/Cas9 protein functions on the principles of a bacterial or archaeal adaptive immunity system that confronts the invading viruses or phages. CRISPR/Cas has been categorized into several types and sub-types such as the class 1 (type I, III, and IV) includes numerous proteins to form complexes, whereas the class 2 (type II, V, and VI) includes only one protein. Among them, Cas9 is the most widely deployed machinery in crop improvement. CRISPR/Cas9 uses specific, designed nucleases to cause double-stranded break (DSB) in DNA (dsDNA). Further, the DSB is repaired by the mechanisms called non-homologous end joining (NHEJ) or homology-directed repair (HDR)/homologus recombination (HR). The NHEJ mechanism is error-prone, allows random small insertions or deletions and substitutions, and probably causes gene knock-out (KO) mutations. The HDR mechanism mostly generates point mutations or deletions caused by gene knock-in (KI), but this method has a very low success rate so far. CRISPR/Cas9 makes breeding much easier by producing gene knock-out mutants for desired traits. Gene knock-in is possible through HDR by providing template DNA with overlapping flanking regions. Nevertheless, CRISPR/Cas9 has many limitations, such as the availability of NGG protospacer adjacent motif (PAM) motifs in the genome sequence. Hence, emphasis has been driven to diversify Cas9 proteins and search for other Cas9 proteins in different bacteria, which have different PAM sequences.

## Strategies and advances in CRISPR/Cas9 technology

### Guide RNA and CRISPR/Cas9 construct design

CRISPR/Cas9 is an RNA-guided genome editing system. The sgRNAs direct Cas9 nuclease to recognize the target DNA sequence to interrupt transcriptional regulation. The gRNA-Cas9 complex identifies the target sequence by gRNA-DNA pairing between the 5’-end sequence of gRNA spacer and one DNA strand (complementary stand of protospacer). Cas9 requires the PAM sequence at the target site. The approximately 20 nucleotide-long gRNA spacer sequence could be readily programmed to target DNA sites with PAM using the online tools available. The freely available online tools for sgRNA design and quality check are CRISPRdirect (https://crispr.dbcls.jp), CRISPR-P (http://cbi.hzau.edu.cn/cgi-bin/CRISPR), CRISPR-PLANT (http://omap.org/crispr/index.html), CRISPR-GE (http://skl.scau.edu.cn/), Breaking-Cas (http://bioinfogp.cnb.csic.es/tools/breakingcas/), CRISPOR.org (http://crispor.org), CRISPR-BETs (Wu et al., 2022) and so on. The MoClo Toolkit (Weber et al., 2011) to assemble sgRNAs constructs and Golden Gate cloning (Engler et al., 2014) protocols have been used in CRISPR/Cas9 genome editing in plants.

The Golden Gate cloning strategy is a very fast and flexible assembly for CRISPR/Cas construct designing (Engler et al., 2014) and applied effectively in tomato (Brooks et al., 2014; Gao et al., 2021; Tran et al., 2021; Do et al., 2022). A new GoldenBraid (GB) assembly of vector construction has shown promising results in tomato (Vázquez-Vilar et al., 2021). An innovative strategy called BioBrick technology consisting of pHNCas9 and pHNCas9HT binary vectors has been devised for functional genomics and genome engineering in plants (Hu et al., 2019). These studies confirm that the CRISPR toolbox can easily carry out single or multi-site editing on multiple genes in tomato.

### *Agrobacterium*-mediated transformation

*Agrobacterium*-mediated transformation is the most commonly used method in CRISPR/Cas9 research in plants including tomato (Lin et al., 2022b; Yang et al., 2022; Yu et al., 2022). Besides, polyethylene glycol (PEG)-based protoplast-mediated transformation and particle bombardment or biolistic methods have also been exhibited in tomato. A notable example elucidates the diverse behavior of Cas9 by engineering QTLs *via* mutagenesis in the *cis*-regulatory regions of the *CLV3 (Clavata3)* gene in tomato (Rodríguez-Leal et al., 2017). Since, sgRNAs and Cas9 gene are eliminated in the next T_1_ generation upon segregation, CRISPR/Cas9 is considered a safe, rapid, and environmentally friendly next-generation breeding technology in crop plants.

### DNA-free protoplast-mediated transformation

*Agrobacterium*-mediated transformation cannot be used for the delivery of Cas9/ribonucleoproteins (RNPs) complexes. Therefore, preassembled Cas9-gRNA RNPs have been directly delivered into the plant cells *via* protoplast-mediated transformation by polyethylene glycol (PEG) fusion or biolistic methods. To remove CRISPR/Cas background, the Cas9-gRNA RNPs system is completely DNA-free and devoid of genetic segregation (Liu et al., 2020b; Thiruppathi, 2022). There are several advantages of Cas9-gRNA RNPs delivery, such as: *i*) avoiding progeny screening by selfing or backcrossing; *ii*) no off-target effects; *iii*) better control over CRISPR/Cas endonuclease; *iv*) direct and easy mutagenesis process after transfection without any lagging phase; and *v*) direct delivery of Cas9-sgRNA assembly or preassembled isolated RNPs into the plant cells. However, RNP’s system has problems like delivery through the plant cells and regeneration from cell-wall-free cells. As a result, protoplast culture protocol is dependent on genotype, species, tissue-specific, and cell wall responses. Thus, minimizing the proportion of off-targets and checking the possibilities of transgene integration is important for enhancing precise Cas9 endonuclease activity.

### CRISPR/Cas9 variants (PAM-free Cas12a, and Cas9-NG/XNG-Cas9)

Cas9 can be used to edit DNA highly efficiently to target gene knock-outs within coding sequences or random changes in non-coding sequences like *cis*-elements for random promoter engineering (Rodríguez-Leal et al., 2017). Further, the elimination of Cas9 components from the plant genome *via* selfing or backcrossing is possible for diploid tomato. The availability of PAM sequences (2-5 bp) is highly lacking in the genome, so it is difficult to edit target genes. Hence, diversity in protospacer adjacent motif (PAM) sequence specificities has become a key requirement of CRISPR/Cas9.

First, PAM-free nucleases have been devised through natural orthologous mining and protein engineering. This can be overcome by Cas9 variants with different PAM-specific sequences, such as Cas12a (class II, type V) requires PAM sequences (5’-TTTN-3’). This is quite useful for the tomato genome, as it has AT-rich sequences and uses Cas12a nuclease. Hence, new variants of Cas9 with diverse sequence specificities of PAM would be greatly useful for the restoration of genetic diversity. The Cas9 variant, a PAM-free Cas12a nuclease, is smaller in size and highly thermostable than Cas9 (Vu et al., 2021). Cas12a (LbCas12a or LbCpf1) from *Lachnospiraceae bacteria* is a new category of the CRISPR system, which is quite analogous to Cas9 and enables editing of AT-rich genomic regions like the 5’ and 3’ UTR and promoter domains. Cas9 endonuclease recognizes G-rich PAM sequences and produces a blunt end cut, whereas Cas12a has a shorter crRNA by 60 nucleotides and no spacer is required, tracrRNA is not required to generate mature crRNA, and recognizes T-rich PAM sequences, producing cohesive ends (Wada et al., 2022). Thus, numerous Cas9 and Cas12a variants are in the process of facilitating sgRNA design and genome editing research.

PAM-free Cas9 has advantages like: i) it can target any sequence; ii) selection of the target site is simple and flexible with more on-target and less off-target; iii) it allows easy placement of base-editing over specific nucleotides; and iv) it is beneficial for multiplexing because only one nuclease targets most genes. Nevertheless, a major limitation of PAM-free nuclease is that the gRNA that is expressed from the DNA constructs would self-target the parent DNA immediately, ultimately leading to unsuccessful results. Another drawback is that a nuclease without a specific PAM would search every sequence in the genome and would take much longer time to find the target sequence, hence increasing the chance of off-targets. Importantly, Cas12a has much scope for PAM-free nuclease activity in plants. Therefore, the development of a nuclease repertoire could be a novel way by which any gene sequence can be targeted by the CRISPR/Cas system.

SpCas9, the most widely used and powerful genome editing tool, requires NGG PAM; thus, its use has been restricted to genomic regions lacking in NGG PAM. The SPCas9 variants xCas9 and Cas9NG have been developed to recognize NG, GAA, and GAT PAMs in human cells. According to Niu et al., 2020, the SpCas9 variants Cas9-NG and XNG-Cas9 can recognize a wide range of NG PAM sites in tomato. Further, a new RNA-guided CRISPR activation (CRISPR-Act3.0) system has been introduced in plants for simultaneous editing of multiple genes based on the deactivated *Streptococcus pyogenes* Cas9 (dSpCas9) and demonstrated in tomato (Pan et al., 2021). This study provides a substantial toolbox for efficient gene activation, an improvement over the CRISPR technology in plants.

### Homology-directed repair and virus-induced genome editing

Precise genome editing is emerging as a promising technology for trait modifications in crops. CRISPR/Cas9 has provided a versatile and efficient option for inducing DSBs in the plant genome. DSB repair by NHEJ can be error-prone and result in partial insertion-deletion that can lead to gene knock-out (KO). On the contrary, DSB repair by HDR requires donor template DNA with homologous flanking sequences that allow gene KI mechanism. NHEJ is the most commonly deployed to develop KO mutants, whereas HDR or KI is promising but has a low success rate in eukaryotic cells. Therefore, the KI mechanism requires more research to handle the technical difficulties in placing the donor templates in the vicinity of the DSB in the recipient plant cells.

*Agrobacterium-*mediated delivery mechanism is most commonly applied in plants, but it is less efficient for HDR-mediated gene editing. Therefore, to address these issues virus-induced genome editing (VIGE) involves plant virus-derived vectors for fast and efficient delivery of sgRNAs (Butler et al., 2016) and has been deployed in tomato. HDR or KI gene editing using the CRISPR/LbCpf1-geminiviral multi-replicon system has increased the editing efficiency by three-fold compared to Cas9-based single-replicon in tomato (Vu et al., 2020b). HR-based knock-in (HKI) frequency has been continuously refined for precision editing in tomato using a combination of Cas9 and a geminivirus replicon, LbCas12a (LbBCpf1), with multi-replicons. Further, HKI could be improved by reengineering temperature-tolerant Cas12a to speed up tomato breeding. Despite a high success rate in plants using geminivirus replicons, the majority of the studies used selection markers with edited alleles. This indicates that geminivirus replicon-based genome editing is still challenging without the use of selection markers for selecting edited lines. Hence, the development of marker-free and selection-free mutants requires further refinement. Thus, CRISPR/Cas9-induced gene replacement *via* the HDR mechanism provides an innovative breeding method in tomato.

### Base/prime editing (Target-AID technology)

The Cas9 nuclease results in DSBs with a high risk of off-target effects. As a result, base editing is a novel CRISPR/Cas9 system approach for editing a single base without DSB or HDR and without donor DNA. Base editing allows mostly transition base changes from C/G to T/A (cytosine base editor, CBE), or A/T to G/C (adenine base editor, ABE), but no CRISPR system has been reported until now for transversion base changes in the plant (Lu et al., 2021; Sretenovic et al., 2021). The ABE has emerged as a boon to gaining gene function. It has been reported that ABE is preferred over CBE in plants because ABE produces fewer off-targets than CBE. Second, the Target activation-induced cytidine deaminase (Target-AID) technology consists of *Petromyzon marinus* cytidine deaminase1 (PmCDA1) fused with nuclease-deficient Cas9 (dCas9) or nCas9 (nickase Cas9 has single-strand DNA cleavage activity). Target-AID using dCas9 results in highly efficient and accurate substitution of C to T, whereas the use of nCas9 causes base substitution and insertion-deletion in the target sites at high efficiency to introduce point mutations (C to T or A to G) without any off-target (Kawaguchi et al., 2021). Third, a recent approach to substitution called prime editing or precise editing has been announced for medium-length DNA using the microhomology-mediated end-joining method. Prime editing technology has been used in animals and monocots, but it is more difficult to apply in dicots. Base or prime editing can easily edit a single base substitution, but problems arise when editing more bases. Several modifications of CRISPR/Cas9 are available for modifying target gene sequences from a single base by base-editors and prime-editors to several kilobases with HKI technique (Vu et al. 2020b). The HKI is a viable option for precise editing. But due to its low frequency and complexity in design, it can be used for modification on a single base or thousands of bases (Vu et al. 2020b). Thus, as a result of a fusion of Cas9 with Target-AID, marker-free mutant plants have been developed with homozygous heritable DNA substitutions, thus demonstrating the feasibility of base editing in tomato improvement.

### Applications of CRISPR/Cas9 in tomato

**Figure 1:**
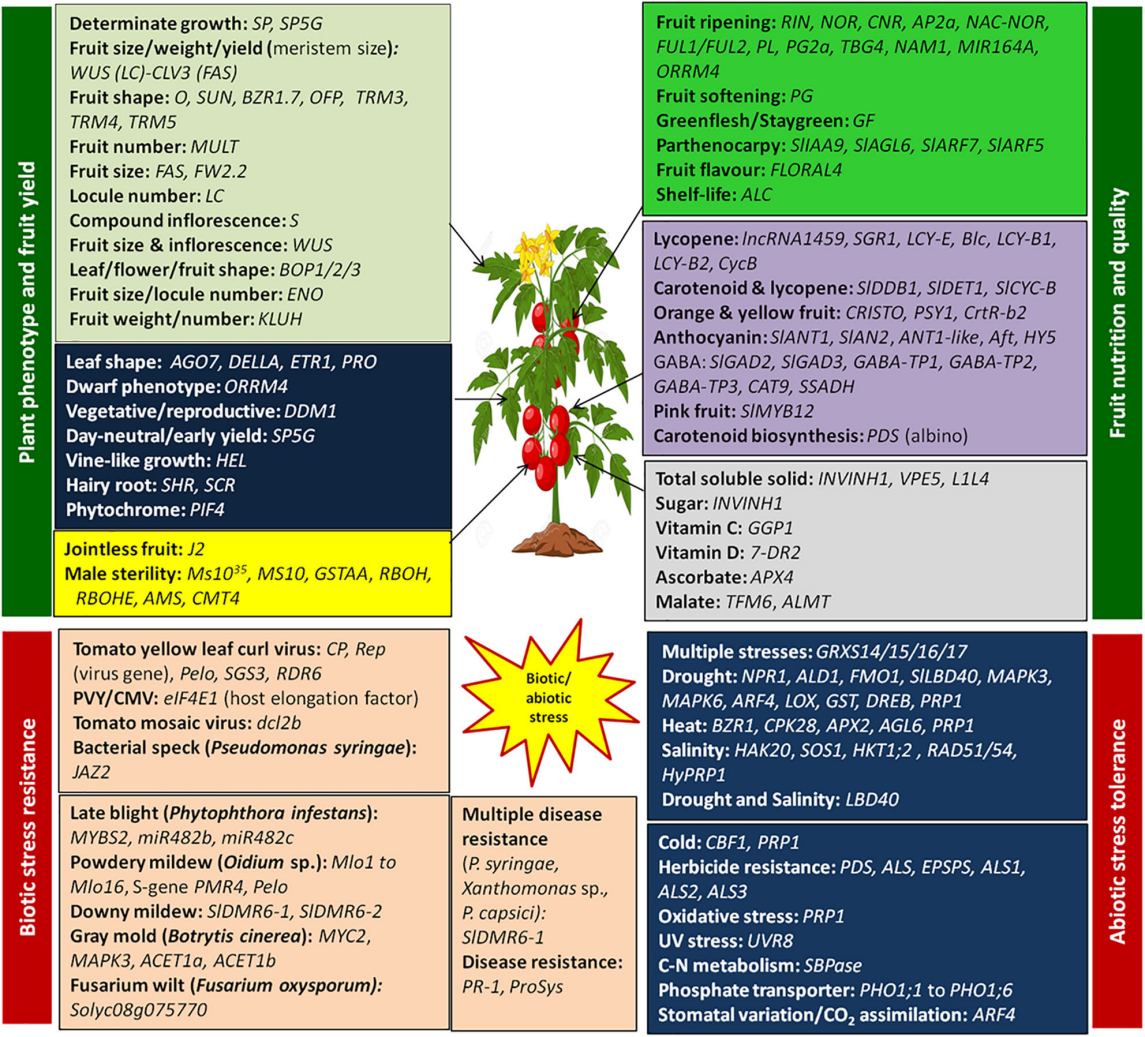
Genome editing of key genes involved in trait modification in plant phenotype, fruit yield, quality, biotic and abiotic stress in tomato.

CRISPR/Cas9 genome editing has revolutionized crop breeding, including tomatoes. CRISPR/Cas9 is the most commonly used genome editing system for precise, efficient, easy, versatile, cost-effective, and targeted gene editing at the desired genomic regions. After the first report in 2013, this technology has been used extensively to edit tomato genotypes. The successful examples of the application of CRISPR/Cas9 genome editing in tomatoes are summarized in **Table 1**. **Figure 1** illustrates various genes demonstrated in tomato traits modification.

**Table 1:** Application of CRISPR/Cas9 technology in tomato improvement for plant architecture, flower, fruit and biotic/abiotic stress resistance-related traits.

Trait	Genotype	Target gene	Gene ID	CRISPR (DNA repair)	Transformation	Genome editing effects	References
Plant architecture, flower and fruit traits							
Plant architecture, lycopene, fruit weight, size, number	*S. pimpinellifolium*	*SP, O*, *MULT*, *FAS* *FW2.2, CNR*, *CycB*	*Solyc06g074350*, *Solyc02g085500*, *Solyc02g077390, Solyc11g071380*, *Solyc02g077920* *Solyc04g040190*	Cas9 (knockout)	*Agrobacterium*	Determinate growth habit (*SP*), increased fruit size (*FAS*), shape (*O*), number (*MULT*), fruit weight (*FW2.2*), and very high lycopene (*CycB*) content	Zsögön et al., 2018
Inflorescence, fruit size, weight, yield	M82, *S. pimpinellifolium*	*WUS/LC*, *CLV3/FAS* *SP*, *S*	*Solyc02g083950,Solyc11g071380*, *Solyc06g074350*, *Solyc02g077390*	Cas9 (knockout)	*Agrobacterium*	Meristem size regulator (*LC-FAS*), increased compound inflorescence, locule number, fruit weight, size, and yield	Rodríguez-Leal et al., 2017
Plant growth habit, fruit	*S. pimpinellifolium*	*SP*, *SP5G*, *CLV3*, *WUS*, *GGP1*	*Solyc06g074350*, *Solyc05g053850*, *Solyc11g071380*, *Solyc02g083950*, *Solyc02g091510*	Cas9 (knockout)	*Agrobacterium*	Determinate growth habit, compact plants, synchronous fruit ripening and enlarged fruit size	Li et al., 2018d
Plant growth habit, flower	M82	*SP5G*	*Solyc05g053850*	Cas9 (knockout)	*Agrobacterium*	Rapid flowering, compact determinate growth habit and early yield	Soyk et al., 2017
Plant growth habit	Red Setter	*SP*, *SP5G*	*Solyc06g074350*, *Solyc05g053850*	Cas9 (Knockin/RNPs)	Protoplast (PEG)	Protoplast-mediated transformation protocols	Liu et al., 2022
Plant regeneration	*S. peruvianum*	*SGS3*, *RDR6*, *PR-1*, *ProSys*, *Mlo1*	*Solyc04g025300*, *Solyc04g014870,Solyc08g075820*, *Solyc09g006005*, *Solyc05g051750*, *Solyc04g049090*	Cas9 (Knockin/RNPs)	Protoplast (PEG)	No chromosomal changes or unintended genome editing sites, and heritable changes	Lin et al., 2022a
Plant regeneration	Micro-Tom	*AGO7*	*Solyc01g010970*	Cas9 (CBEs/ABEs)	Protoplast (PEG)	Successful application of cytosine base editors (CBEs) and adenine base editors (ABEs) in rice, tomato and poplar	Sretenovic et al., 2021
Plant phenotype	Micro-Tom	*PDS*, *PIF4*	*Solyc03g123760*, *Solyc07g043580*	Cas9 (knockout)	*Agrobacterium*	High frequencies of homozygous and biallelic albino mutants	Pan et al., 2016
Plant phenotype	Ailsa Craig and Micro-Tom	*PDS*	NM_001247166	Cas9 (knockout)	*Agrobacterium*	Albino mutants	Li et al., 2018c
Plant regeneration	Red Setter, Ailsa Craig, M82, Moneymaker	*SP*, *SP5G*	*Solyc06g074350*, *Solyc05g053850*	Cas9 (RNPs)	Protoplast (PEG-Ca^2+^)	Improved protoplast isolation and shoot regeneration	Liu et al., 2022
Plant regeneration	*Solanum* spp.	*SHR*, SCR	*Solyc02g092370, Solyc10g074680*	Cas9 (knockout)	*A.* *rhizogenes*	Hairy root transformation	Ron et al., 2014
Dwarf plant	Moneymaker	*PRO*	*Solyc11g011260*	Cas9 (knockout)	*Agrobacterium*	Gibberellins-responsive dominant dwarf mutant with loss of function and deletion in DELLA allele	Tomlinson et al., 2019
Leaf shape	M82	*AGO7*	*Solyc01g010970*, *Solyc07g021170*, *Solyc08g041770*, *Solyc12g044760*	Cas9 (knockout)	*Agrobacterium*	Change in leaf shape from compound flat to needle like leaves	Brooks et al., 2014
Leaf shape	Micro-Tom	*DELLA*, *ETR1*	*Solyc11g011260*, *Solyc12g011330*	Cas9 (base editing, Target-AID)	*Agrobacterium*	Reduced serrated leaflets and insensitivity to ethylene	Shimatani et al., 2017
Male sterility	Micro-Tom	*Ms10^35^*, *GSTAA*	*Solyc02g079810*, *Solyc02g081340*	Cas9 (knockout)	*Agrobacterium*	Male-sterile line with a green hypocotyl or trichome density	Liu et al., 2021a
Male sterility	KS-13	*MS10*	*Solyc02g079810*	Cas9 (knockout)	*Agrobacterium*	Male sterile line	Jung et al., 2020
Male sterility	Alisa Craig	*RBOH, RBOHE*	*Solyc01g099620*, *Solyc07g042460*	Cas9 (knockout)	*Agrobacterium*	Male sterile line	Dai et al., 2022
Male sterility	Ailsa Craig	*AMS*	QDO73362.1	Cas9 (knockout)	*Agrobacterium*	Non-viable pollens	Bao et al., 2022
Male sterility	Ailsa Craig	*CMT4*	*Solyc08g005400*	Cas9 (knockout)	*Agrobacterium*	Suppressed pollen tube growth	Guo et al., 2022
Jointless fruit	M82	*J2*	*Solyc12g038510*,	Cas9 (knockout)	*Agrobacterium*	Jointless inflorescence, large sepals	Roldan et al., 2017
Leaf, flower, fruit shape	M82	*BOP1/2/3* (*TMF*)	*Solyc04g040220*, *Solyc10g079460*, *Solyc10g079750*	Cas9 (knockout)	*Agrobacterium*	Defects in fruit shape and altered leaf	Xu et al., 2016
Fruit size, locule number	*S. pimpinellifolium*	*ENO*	*Solyc03g117230*	Cas9 (knockout)	*Agrobacterium*	Increased fruit locule number, fruit size and yield	Yuste-Lisbona et al., 2020
Fruit weight, number	*S. pimpinellifolium*	*KLUH* (*CYP78A* family)	M9 SNP	Cas9 (knock-out)	*Agrobacterium*	Increased fruit weight and decreased number of small fruits	Li et al., 2022b
Fruit size	Ailsa Craig	*SUN*, *BZR1.7*	*Solyc10g079240*, *Solyc10g076390*	Cas9 (knockout)	*Agrobacterium*	Elongated fruit shape	Yu et al., 2022
Fruit size	*S. lycopersicum, S. pimpinellifolium*	*TRM3/4/5*, *OFP*	*Solyc07g008670, Solyc10g076180*	Cas9 (knockout)	*Agrobacterium*	Slightly flatter fruit	Wu et al., 2018
Fruit ripening	Micro-Tom	*MIR164A* (targets: *NAM2, NAM3*)	*Solyc03g115850, Solyc06g069710*	Cas9 (knockout)	*Agrobacterium*	Accelerated fruit ripening	Lin et al., 2022b
Fruit ripening	Ailsa Craig	*RIN*	*Solyc05g012020*	Cas9 (knockout)	*Agrobacterium*	Partial ripening and moderate red pigmentation	Ito et al., 2015; Ito et al., 2017; Ito et al., 2020
Fruit ripening	Ailsa Craig	*NOR*, *CNR*	*Solyc10g006880* *Solyc02g077920*	Cas9 (knockout)	*Agrobacterium*	Delayed ripening by 2-3 days	Gao et al., 2019
Fruit ripening	Moneyberg, Ailsa Craig	*AP2a*, *NAC-NOR, FUL1*, *FUL2*	*Solyc03g044300*, *Solyc10g006880*, *Solyc06g069430, Solyc03g114830*	Cas9 (knockout)	*Agrobacterium*	Early fruit ripening but not fully ripen	Wang et al., 2019a
Fruit ripening	Ailsa Craig	*PL*, *PG2a, TBG4*	*Solyc03g111690*, *Solyc10g080210, Solyc12g008840*,	Cas9 (knockout)	*Agrobacterium*	Influenced fruit firmness, weight and color	Wang et al., 2019a
Fruit ripening	Ailsa Craig	*NAM1*	*Solyc06g060230*	Cas9 (knockout)	*Agrobacterium*	Regulates fruit ripening	Gao et al., 2021
Fruit ripening	Moneyberg, Ailsa Craig	*FUL1*, *FUL2*	*Solyc06g069430, Solyc03g114830*	Cas9 (knockout)	*Agrobacterium*	Independent/overlapping functions in fruit ripening	Wang et al., 2019a
Fruit ripening	Micro-Tom	*ORRM4*	*-*	Cas9 (knockout)	*Agrobacterium*	Involved in RNA editing of transcripts	Yang et al., 2017
Parthenocarpy	Micro-Tom, Ailsa Craig	*IAA9*	*Solyc04g076850*	Cas9 (knockout)	*Agrobacterium*	Leaf shape changes and parthenocarpic	Ueta et al., 2017
Parthenocarpy	MP-1	*AGL6*	*Solyc01g093960*	Cas9 (knockout)	*Agrobacterium*	Parthenocarpic fruit and heat stress tolerance	Klap et al., 2017
Fruit flavor	F_6_ (RILs)	*FLORAL4*	*Solyc04g063350*	Cas9 (knockout)	*Agrobacterium*	Increased phenylalanine volatile	Tikunov et al., 2020
Lycopene	–	*lncRNA1459*	*KT963310*, *KT963311*	Cas9 (knockout)	*Agrobacterium*	Reduced ethylene and lycopene	Li et al., 2018a
Lycopene	Ailsa Craig	*SGR1, LCY-E*, *Blc*, *LCY-B1, LCY-B2*	*DQ100158*, *EU533951*, *XM_010313794*, *EF650013*, *AF254793*	Cas9 (knockout)	*Agrobacterium*	Increased lycopene	Li et al., 2018b
Lycopene	*S. pimpinellifolium*	*CycB*	*Solyc04g040190*	Cas9 (knockout)	*Agrobacterium*	High lycopene content	Zsögön et al., 2018
Carotenoid, chlorophyll, *B. cinerea*	Moneymaker, San Marzano	*GF*	*Solyc08g080090*	Cas9 (knockout)	*Agrobacterium*	high carotenoids, chlorophylls and *Botrytis cinerea* resistance	Gianoglio et al., 2022
Yellow fruit	Red Setter	*Psy1*, *CrtR-b2*	*Solyc03g031860*, *Solyc03g007960*	Cas9 (knockout)	*Agrobacterium*	Yellow flesh color fruit	D’Ambrosio et al., 2018
Carotenoid	Periyakulam 1 (PKM1)	*CRTISO*	*AF416727*	Cas9 (knockout)	*Agrobacterium*	Complete knockout of protein function	Jayaraj et al., 2021
Pink fruit	Inbred lines	*MYB12*	*Solyc01g079620*	Cas9 (knockout)	*Agrobacterium*	Pink color fruit	Deng et al., 2018
Orange and yellow fruit	Micro-Tom	*CRISTO*, *PSY1*	*-*	Cas9 (knockin/geminiviral replicon)	*Agrobacterium*	High editing efficiency	Dahan-Meir et al., 2018
Yellow fruit	M82, *S. pimpinellifolium*	*PSY1*	*Solyc03g031860*	Cas9 (knockin)	*Agrobacterium*	Yellow fruit	Hayut et al., 2017
Carotenoid, lycopene	Micro-Tom	*DDB1, DET1*, *CYC-B*	*Solyc02g021650*, *Solyc01g056340*, *Solyc06g074240*	Cas9 (Target-AID base editing)	*Agrobacterium*	Increased carotenoid, lycopene, and β-carotene content	Hunziker et al. (2020)
Anthocyanin	Micro-Tom	*ANT1*	*Solyc10g086260*	Cas9 (Geminivirus replicon)	*Agrobacterium*	High anthocyanin content	Cˇermák et al., 2015; Vu et al., 2020b
Anthocyanin	Indigo Rose	*AN2*, *ANT1*, *ANT1-* *like*, *R2R3-MYB TF AN2-like/Anthocyanin fruit (Aft)*	*Solyc10g086250, Solyc10g086260, Solyc10g086270*, *Solyc10g086290*	Cas9 (knockout)	*Agrobacterium*	Decreased anthocyanin content	Yan et al., 2020; Zhi et al., 2020
Anthocyanin	Indigo Rose	*HY5*	*Solyc08g061130*	Cas9 (knockout)	*Agrobacterium*	Decreased anthocyanin	Qiu et al., 2019
GABA	Micro-Tom	*GAD2*, *GAD3*	B1Q3F1 FB1Q3F2	Cas9 (knockout)	*Agrobacterium*	Increased GABA content	Nonaka et al., 2017
GABA	Ailsa Craig and Micro-Tom	*GABA-TP1, GABA-TP2*, *GABA-TP3, CAT9*, *SSADH*	AY240229, AY240230, AY240231, XM_004248503, NM_001246912	Cas9 (knockout)	*Agrobacterium*	Increased GABA content	Li et al., 2018c
Total soluble solid (TSS)	M82	*INVINH1*, *VPE5*	*Solyc12g099200*, *Solyc12g095910*	Cas9 (knockout)	*Agrobacterium*	Increased glucose, fructose, and TSS contents	Wang et al., 2021a
Sugar	Suzukoma	*INVINH1*	*Solyc12g099200*	Cas9 (knockout, Target-AID base editing)	*Agrobacterium*	High sugar content	Kawaguchi et al., 2021
Vitamin D	Moneymaker	*7-DR2*	*Solyc06g074090*	Cas9 (knockout)	*Agrobacterium*	Increased 7-dehydrocholesterol (7-DHC) level	Li et al., 2022a
Ascorbate	Micro-Tom	*APX4*	*Solyc06g005150*	Cas9 (knockout)	*Agrobacterium*	Increased ascorbate content	Do et al., 2022
Malate	*S. lycopersicum*, *S. pimpinellifolium*, *S.l.* var *cerasiforme*	*TFM6*, *ALMT*	*Solyc06g072910*, *Solyc06g072920*	Cas9 (knockout)	*Agrobacterium*	Reduced malate content	Ye et al., 2017
Shelf-life	M82	*ALC*	FJ404469	Cas9 (knockin)	*Agrobacterium*	Extended shelf-life	Yu et al., 2017
Fruit softening	Micro-Tom	*PG*	*solyc10g080210*	Cas9 (knockout)	*A. rhizogenes*	Delayed fruit softening	Nie et al., 2022
*Biotic stress resistance*							
TYLCV	Moneymaker	*CP, Rep* (virus gene)	*-*	Cas9 (knockout)	*Agrobacterium*	Resistance	Tashkandi et al., 2018
TYLCV	Moneymaker	*rgsCaM* promoter	*-*	Cas9 (knockout)	*Agrobacterium*	Efficiency of an inducible promoter	Faal et al., 2020
TYLCV, Powdery mildew	BN-86	*Pelo*, *Mlo1*	*Solyc04g009810*, *Solyc04g049090*	Cas9 (knockout)	*Agrobacterium*	High resistance	Pramanik et al., 2021
ToMV	Ailsa Craig	*DCL2b*	–	Cas9 (knockout)	*Agrobacterium*	Resistance	Wang et al., 2018
Bacterial speck (*Pseudomonas syringae*)	Moneymaker	*JAZ2*	*Solyc12g009220*	Cas9 (knockout)	*Agrobacterium*	Resistance	Ortigosa et al., 2019
Late blight (*Phytophthora infestans*)	Alisa Craig	*MYBS2*	*Solyc04g008870*	Cas9 (knockout)	*Agrobacterium*	High resistance	Liu et al., 2021b
Powdery mildew (*Oidium* sp.)	Moneymaker	*Mlo1*	*Solyc04g049090*	Cas9 (knockout)	*Agrobacterium*	Resistance	Nekrasov et al., 2017
Powdery mildew	Moneymaker	*PMR4*	*Solyc07g053980*	Cas9 (knockout)	*Agrobacterium*	Reduced resistance	Martínez et al., 2020
Downy mildew	FL8000	*DMR6-1, DMR6-2*	*Solyc03g080190*, *Solyc06g073080*	Cas9 (knockout)	*Agrobacterium*	High resistance	Thomazella et al., 2021
Multiple diseases	FL8000	*DMR6-1*	*Solyc03g080190*	Cas9 (knockout)	*Agrobacterium*	Disease resistance to *P. syringae*, *P. capsici*, and *Xanthomonas* spp.	Thomazella et al., 2016
Fusarium wilt	76R, *rmc*	*-*	*Solyc08g075770*	Cas9 (knockout)	*Agrobacterium*	Resistance	Prihatna et al., 2018
Gray mold (*Botrytis cinerea*)	Micro-Tom, Ailsa Craig	*MYC2*, *MAPK3*	*-*	Cas9 (knockout)	*Agrobacterium*	Decreased disease resistance to *B. cinerea*	Zhang et al., 2018; Shu et al., 2020
PVY, CMV	Inbred line S8	*eIF4E1* (host gene)	–	Cas9 (knockout)	*Agrobacterium*	PVY and CMV resistant mutants	Atarashi et al., 2020
*Abiotic stress tolerance*							
Multiple stresses	Rubion	*GRXS14/15/16/17*	*Solyc02g082200, Solyc06g067960*, *Solyc09g005620, Solyc02g078360*	Cas9 (knockout)	*Agrobacterium*	Increased tolerance to heat, chilling, drought, heavy metal toxicity and nutrient deficiency	Kakeshpour et al., 2021
Drought	Ailsa Craig	*NPR1*	KX198701	Cas9 (knockout)	*Agrobacterium*	Reduced drought tolerance	Li et al., 2019
Drought	Condine Red	*ALD1*, *FMO1*	*Solyc11g044840*, *Solyc07g04243*	Cas9 (knockout)	*Agrobacterium*	Increased drought tolerance	Wang et al., 2021b
Drought	Micro-Tom	*LBD40*	*Solyc02g085910*	Cas9 (knockout)	*Agrobacterium*	Increased drought tolerance	Liu et al., 2020a
Drought	Ailsa Craig	*MAPK3*	AY261514	Cas9 (knockout)	*Agrobacterium*	Reduced drought tolerance	Wang et al., 2017
Heat	Condine Red	*BZR1*	*Solyc04g079980*	Cas9 (knockout)	*Agrobacterium*	Reduced heat tolerance	Yin et al., 2018
Heat	Condine Red	*CPK28*, *APX2*	*Solyc02g083850, Solyc06g005150*	Cas9 (knockout)	*Agrobacterium*	Improved thermotolerance	Hu et al., 2021
Heat	MP-1	*AGL6*	*Solyc01g093960*	Cas9 (knockout)	*Agrobacterium*	Seedless fruit under heat stress	Klap et al., 2017
Cold	Ailsa Craig	*CBF1*	AAS77820	Cas9 (knockout)	*Agrobacterium*	Reduced chilling tolerance	Li et al., 2018e
Salinity	*S. lycopersicum, S. pimpinellifolium*	*HAK20*, *SOS1*	*Solyc04g008450, Solyc11g044540*	Cas9 (knockout)	*Agrobacterium*	Increased salt sensitivity	Wang et al., 2020; Wang et al., 2021c
Salinity	Hongkwang	*HKT1;2* *RAD51/54*	*Solyc07g017540*, *Solyc04g056400*	Cas12a/ LbCpf1 (Geminivirus replicon)	*Agrobacterium*	Increased salt tolerance	Vu et al., 2020b
Salinity	Hongkwang	*HyPRP1*	*Solyc12g009650*	Cas9 (knockout)	*Agrobacterium*	Increased salt tolerance	Tran et al., 2021
C-N metabolism	Micro-Tom	*SBPase*	*Solyc05g052600*	Cas9 (knockout)	*Agrobacterium*	Optimal growth, carbon assimilation and nitrogen metabolism	Ding et al., 2018
Phosphate transporter	Micro-Tom	*PHO1;1*, *PHO1;2*, *PHO1;3*, *PHO1;4*, *PHO1;5*, *PHO1;6*	*Solyc09g090360* *Solyc08g068240, Solyc05g010060*, *Solyc05g013180, Solyc02g088220, Solyc02g088230*	Cas9 (knockout)	*Agrobacterium*	High anthocyanin and phosphate content	Zhao et al., 2019
Herbicide resistance	WVA106	*ALS*	*Solyc03g044330*	Cas9 (base editing)	*Agrobacterium*	Resistance (Chlorsulfuron)	Veillet et al., 2019
Herbicide resistance	Micro-Tom	*PDS*, *ALS*, *EPSPS*	*Solyc03g123760*, *Solyc06g059880, Solyc03g044330*, *Solyc01g091190*	Cas9 (knockout)	*Agrobacterium*	Resistance	Yang et al., 2022
Herbicide resistance	–	*ALS1*, *ALS2*, *ALS3*	*Solyc03g044330*, *Solyc07g061940, Solyc06g059880*	Cas9 (knockin)	*Agrobacterium*	Resistance (Chlorsulfuron)	Danilo et al., 2019

7-DR2 (7-Dehydrocholesterol reductase), AGL6 (Agamous-like 6), AGO7 (Argonaute7), ALC (Alcobaca), ALD1 (AGD2-like defense response protein), ALMT (Al-activated malate transporter),ALS1 (Acetolactate synthase 1), AMS (Aborted microspores), AN2 (Anthocyanin2), ANT1 (Anthocyanin 1), AP2a (Apetala2a TF), APX2/4 (Ascorbate peroxidase2/4), Blc (Beta-lycopene cyclase),BOPs (Blade-on-petiole), BZR1 (Brassinazole-resistant 1), CRTISO (Carotenoid isomerise), CAT9 (Cationic amino acid transporter 9), CBF1 (C-repeat/dehydration responsive element binding factor1), CLV3 (Clavata3), CMT4 (Chromomethylase), CNR (SBP-box colorless non-ripening), CPK28 (Calcium-dependent protein kinase28), CP (Coat protein), CrtR-b2 (Beta-carotene hydroxylase 2),CRTISO (Central role of carotenoid isomerase), CYC-B/CycB (Lycopene beta cyclase), DDB1 (DNA damage UV binding protein 1), DELLA (Aspartic acid–glutamic acid–leucine–leucine–alanine),DCL2b (Dicer-like 2b), DET1 (Deetiolated1), DMR6 (Downy mildew resistance 6), EJ2 (Enhancer-of-jointless2), ENO (Excessive number of floral organs), EPSPS (5-Enolpyruvylshikimate-3-phosphate synthase), ETR1 (Ethylene receptor 1), FASCIATED (FAS), FMO1 (Flavin-dependent monooxygenase), FUL1/2 (Fruitfull), GABA-TP1 (Pyruvate-dependent g-aminobutyric acidtransaminase 1), GAD2 (Glutamate decarboxylase 2), GAD3 (Glutamate decarboxylase 3), GF (Greenflesh/Staygreen), GGP1 (GDP-l-galactose phosphorylase1), GRXS (CGFS-type glutaredoxin),GSTAA (Glutathione S-transferase), HAK20 (High-affinity K+ 20), HKT1;2 (High-affinity potassium transporter 1;2), HY5 (elongated hypocotyl5), HyPRP1 (Hybrid proline-rich protein 1), IAA9(Auxin-induced 9), INVINH1 (Invertase inhibitor 1), J2 (Jointless-2), JAZ2 (Jasmonate zim domain), LBD40 (Lateral organ boundaries domain40), LOCULE NUMBER (LC), LCY-B1 (Lycopene bcyclase1), LCY-B2 (Lycopene b-cyclase 2), LCY-E (Lycopene e-cyclase), LIN (Long inflorescence), MAPK3 (Mitogen activated protein kinase 3), MIR164A (MicroRNA164A), TFAM1/TFAM2(Mitochondrial transcription factor A), Mlo1 (Mildew resistance locus o 1), MS10 (Male sterile 10), Ms1035 (Male sterile 1035), MULT (Multiflora), MYBS2 (MYB transcription factor S2), MYC2(Basic helix–loop–helix transcription factor), NAC (NAM-ATAF-CUC), NAM1/2/3 (No apical meristem1/2/3), NAC-NOR (NAC TF non-ripening), NOR (Non-ripening), NOR-like1 (Non-ripeninglike1), NPR1 (Nonexpressor of pathogenesis-related gene 1), O (ovate), ORRM4 (organelle RNA recognition motif-containing protein4), PDS (Phytoene desaturase), OFP (OVATE family protein),PG (Polygalacturonase), PG2a (polygalacturonase 2a), PHO1 (Phosphate 1), PIF4 (Phytochrome interacting factor 4), PL (pectate lyase), PMR4 (Powdery mildew resistance 4), PR-1 (Pathogenesisrelatedprotein-1), PRO (Procera), ProSys (Prosystemin), PSY1 (Phytoene synthase 1), RAD51/54 (DNA repair and recombination protein51/54), RBOH/RBOHE (Respiratory burst oxidasehomolog), RDR6 (RNA ald1dependent RNA polymerase 6), REP (Replicase), RIN (Ripening inhibitor), RMC (Reduced mycorrhizal colonization), SBPase (Sedoheptulose-1,7-bisphosphatase), S(Compound inflorescence), SCR (Scarecrow), SHR (Shortroot), SGR1 (Stay-green 1), SGS3 (Suppressor of gene silencing 3), SOS1 (Salt overly sensitive 1), SP (Self pruning), SP5G (Self pruning 5G),SSADH (Succinate semialdehyde dehydrogenase), Target-AID (Target activation-induced cytidine deaminase), TBG4 (b-galactanase), TFM6 (Tomato fruit malate on chromosome6), TMF(Terminating flower), ToMV (Tomato Mosaic virus), TRM3/4/5 (TONNEAU1 Recruiting Motif3/4/5), VPE5 (Vacuolar processing enzyme5), and WUS (Wuschel).RNPs (ribonucleoproteins), PEG (polyethylene glycol), Target-AID (Target Activation Induced Cytidine Deaminase), NHEJ (Homologous-End-Joining), KO (gene knock-out), KI (gene knock-in), HDR (homology-directed repair), HR (homologous recombination), HKI (HR-based KI).

### Plant architecture, flower, and fruit traits

CRISPR/Cas9 technology has been deployed successfully in tomato for plant architecture, flower and fruit traits (**Table 1**). In the early domestication process, small-fruited cherry tomato led to the development of large-fruited modern tomato cultivars. During this process a number of traits were evolved and now it has been proven by CRISPR/Cas9 genome editing. Cas9-based genome engineering has been applied in tomato for yield and its related traits such as a determinate plant growth habit caused by the flowering repressor *SP* (*Self pruning*) gene, a 3-fold increase in fruit size by the gene *FAS* (*fasciated*), an oval shape the *ovate* (*O*) gene, a 10-fold increase in fruit number by the gene *MULT* (*multiflora*), an increased fruit weight by the gene *FW2.2* (*fruit weight*), and a very high lycopene content that has been improved by 500% (*CYC-B/CycB*). Moreover, many other traits have also been edited such as male sterility for hybrid seed production *via* co-knockouts of the *Ms1035 (Male Sterile 1035)*, *GSTAA (Glutathione S-Transferase)* genes (Liu et al., 2021a), *J2 (Jointless-2)* gene for mechanical harvesting (Roldan et al., 2017), and pectin degradation in ripening tomato (Wang et al., 2019c).

The essential role of the *RIN (ripening inhibitor)* gene encoding the MADS-box transcription factor in fruit ripening is well known, as evidenced by various researchers (**Table 1**). RIN gene-defective mutants yielded partially ripened fruits, whereas heterologous mutants yielded fully ripened red fruits, confirmed its role in fruit ripening in tomato (Ito et al., 2015; Ito et al., 2017; Ito et al., 2020). The roles of the genes *SGR1 (Stay-Green1)*, *LCY-E (Lycopene e-cyclase), Blc (Beta-lycopene someri)*, and LC*Y-B1/LCY-B2 (Lycopene b-cyclase 1/2)* have been illustrated in the carotenoid metabolic pathways by using CRISPR/Cas9 gene knockout mechanism. Lycopene content in the mutants was increased by about 5.1-fold along with other advantages such as high efficiency, rare off-target mutations, and stable heredity (Li et al., 2018b). The *CYC-B/CycB (Lycopene beta someri)* gene-mediated Cas9 editing increased the lycopene content in tomato (Zsögön et al., 2018). Cas9-mediated editing of the *AGL6 (Agamous-like 6)* gene resulted in parthenocarpic fruit development under heat stress conditions in tomato (Klap et al., 2017). A large number of traits have been modified using CRISPR/Cas9 in tomato for fruit color (yellow, pink and purple). Increased GABA content (7–15 folds) in fruits was obtained by CRISPR/Cas9 editing of *GAD2/3 (Glutamate Decarboxylase 2/3)* genes (Nonaka et al., 2017). High total soluble solids (TSS) content is very important in tomato varieties for processing purposes. Recently, Wang et al. (2021a) regenerated CRISPR gene knockout mutants of *INVINH1* and *VPE5* genes with significantly increased levels of glucose, fructose, and TSS content.

### Biotic and abiotic stress resistance/tolerance traits

Tomato is affected seriously by various biotic (disease and insect-pest) and abiotic (heat, drought, cold, salinity) stresses. To overcome these problems, in the recent years many traits have been edited by CRISPR/Cas9 tools (**Table 1**). Importantly, tomato yellow leaf curl virus (TLCV) is the most devastating disease of tomato. CRISPR/Cas9 has been successfully deployed to develop TYLCV resistant plants. High resistance to TYLCV (*Ty-5*) and powdery mildew diseases were developed *via* Cas9 editing of the *Pelo* and *Mlo1* genes (Pramanik et al., 2021). Further, multiple disease-resistant mutants have been regenerated *via* CRISPR/Cas9 editing of *DMR6-1 (Downy mildew resistance 6-1)* for *P. syringae, P. capsici*, and *Xanthomonas* spp. (Thomazella et al., 2016; Thomazella et al., 2021). They showed that mutants displaying up-regulation of *DMR6-1* after pathogen infection has enhanced disease resistance, which was correlated with increased salicylic acid (SA) (Thomazella et al., 2016; Thomazella et al., 2021). Late blight (*Phytophthora infestans*) resistant mutants were also developed by CRISPR/Cas9 editing of the *MYBS2 (MYB transcription factor S2)* gene (Liu et al., 2021b).

Heat stress is one of the most serious issues in tomato cultivation in climate change scenarios. Several abiotic stresses related traits have been altered by CRISPR/cas9 (**Table 1**). CRISPR/Cas9 editing of the *CPK28 (Calcium-dependent protein kinase28)* gene targeting *APX2 (Ascorbate peroxidase 2)* improved thermotolerance in tomato (Hu et al., 2021). CRISPR/Cas9 of *GRXS14/15/16/17 (CGFS-type glutaredoxin 14/15/16/17)* genes showed increased tolerance to multiple abiotic stresses such as heat, chilling, drought, heavy metal toxicity, and nutrient deficiency (Kakeshpour et al., 2021). Salinity stress tolerance was detected in Cas9 mutants of genes like *HKT1;2 (High-affinity potassium transporter1;2)*, *RAD51/54 (DNA repair and recombination protein* 51/54) (Vu et al., 2020b), and *PR-1 (Pathogenesis-related protein 1)* (Tran et al., 2021). Taken together, a large number of traits have been modified in tomato applying CRISPR/Cas9 (**Table 1**).

## Concluding remarks

The CRISPR/Cas system is a powerful technology for next-generation breeding of tomato. An immense development has been observed in CRISPR/Cas research, such as protoplast-mediated sgRNAs/RNPs transformation, the PAM-free system, dCas9-mediated epigenome modification and targeted base/prime editing. However, many questions remain unanswered, like the missing link connecting interference, adaptation in primed spacer acquisition, and the mechanism behind the horizontal transfer of CRISPR/Cas. Still, several challenges are involved in CRISPR/Cas. First, the generation of off-targets is the major concern in genome editing, which can be reduced considerably using the Cas9 variant (Cas12a) with diverse PAM sequence specificities and base or prime editing. Second, off-target mutants are reduced by increasing the specificity of Cas9 cleavage *via* the induction of double nickage mutants or truncation of the gRNA. It is also important to reduce the problems caused by the low frequency of HDR in plants. Third, multiplex editing is an efficient strategy for the simultaneous dissection of multiple genes, proteins, and metabolites at specific precision. Fourth, problems are associated with the selection of a large number of bases, which is possible with HKI. Fifth, dealing with in-planta transformation issues in *Agrobacterium*-mediated (Maher et al., 2020) or nanoparticle-mediated (Cunningham et al., 2018) transformation, and so on. Altogether, an appropriate sgRNA, Cas array, efficient transformation system, and phenotype without off-targets are the key considerations of CRISPR/Cas9.

Innovations in Cas9 offer precise editing of targeted genes and even simultaneous editing of multiple genes by multiplexing to accelerate breeding at reduced costs. Diversity in CRISPR/Cas toolbox is required to mediate the catalytic activities of CRISPR/Cas along with environmentally friendly protocols for efficient delivery of Cas9. New CRISPR/Cas tools have created more efficient and precise editing *via* base editing, prime editing, and HKI. Allele introgression *via* targeted mutagenesis, as well as base editing and HKI in tomato, has become easier and less time-consuming. The discovery of programmable Cas9 variant and Cas12a nucleases has widened more scope without any off-targets. The use of a multiplexing approach with a robust CRISPR/Cas9 array having single or multiple sgRNAs or RNPs targeting conserved sequences of target genes and protoplast-mediated transformation could be deployed for precision editing. Homozygous edited lines can be obtained through haploid-inducer-based genome editing. Cas9 components can be removed *via* genetic segregation, and transgene-free genome-edited mutants can be obtained to meet regulatory requirements. More importantly, public awareness is necessary for the popularity of genome-edited plants, which do not contain any foreign genes. Hence, the regulatory process needs to be harmonized in public for genome-edited mutants. There is no concern about biosafety regulation in genome-edited plants because they are free of foreign genes, particularly site-directed nucleases (SDN1 and SDN2), and the vector backbone is removed through genetic segregation *via* backcrossing. The United States Department of Agriculture provides an exemption for these genome-edited crops, e.g., mushroom and corn; on the contrary, the Court of Justice of the European Union considered genome-edited plants under the same transgenic category. Further, the USA has released six virus-resistant genome-edited tomatoes. Genome-edited plants could be accepted as transgene-free (non-GM) by the public and regulatory bodies, which would open more avenues for the application of CRISPR/Cas in tomatoes. Taken together, there is a tremendous potential to deploy CRISPR/Cas9 for trait modifications in tomato.

## Author contributions

JT: conceptualized and wrote the manuscript; AS and TB critically edited the manuscript. All authors contributed to the article and approved the submitted version.
